# Progress in Surgery

**Published:** 1898-01-08

**Authors:** 


### PROGRESS IN SURGERY.

### GENERAL SURGERY.

(*Continued from page 244.*)

**Fractures and Dislocations.—Lower Extremity.**—Dr. N. M. Shaffer[38] again calls attention to his method of treating ununited fractures of the neck of the femur. It consists in the application of a long hip-splint, with a surcingle round the hips, and in the application of direct pressure over the fragments by means of a tourniquet. This pressure should not be very great, except when the patient is moved in the bed. It is important to slightly abduct the limb, as this favours good approximation of the fragments. In recent fractures of the neck of the femur, Dr. John Ridlon[39] thinks that, if there is impaction, the fragments should not be intentionally disengaged, but otherwise one should not consider that proper approximation has been secured until the antero-posterior, lateral, and rotary deformity has been corrected, and the limb restored to nearly its normal length. Thomas's long hip-splint should then be applied, with adhesive plaster on both sides of the leg, and buckled to the ring. As no shoulder-straps are employed, the weight of the splint produces a certain amount of constant traction. In his experience this arrangement has proved to be a very convenient one in private practice, as the patient can be very readily moved about in bed without harm to the fracture. Dr. G. S. Brown[40] recalls the attention of the profession to the old but much neglected Hodgens' splint for fractures of the thigh. It is somewhat remarkable that so useful and simple a device as this should have fallen into disuse, or, rather, should fail to be as widely adopted as it deserves. To his mind, Hodgens' splint is scientific and gives no pain, and, compared with Buck's apparatus, the long splint, and plaster dressings are little short of crude and barbarous. In the treatment of fractures of the patella, Dr. Lejars[41] has employed "circling" in seven cases. This method appears to him to be indicated principally in cases in which the lower fragment is so small that it could barely be perforated once, and wiring, therefore, a difficult matter. A second variety of cases for this treatment is direct fracture with many fragments, wiring being then also impossible. Dr. Poirier[42] thinks that the only case in which this operation is suitable is when the patella is peculiarly friable. The treatment of fractures of the patella cannot be uniform. When the bone is firm, the fragments should be wired, but when it is friable recourse should be had to circling. Dr. Peyrot[43] is of opinion that circling "is the best method in respect of firmness and solidity," while Dr. Lucas Championnière regards wiring as the superior procedure to all others. The latter surgeon has performed 50 operations on the patella, with only four deaths, one of which was due to chloroform. He gives the circumstances under which death ensued in the other three cases. He is in favour also of early movement, his patients beginning to walk on the eighteenth, fourteenth, sometimes even on the tenth day. Mr. W. A. Lane[44] has lately written again upon the subject of the open treatment of simple fractures of the tibia. He describes in detail the methods he adopts in several forms of fracture, and reproduces some skiagrams of patients after operation. Three examples of compound fracture of the lower third of the leg are related by Dr. M. B. Long.[45] In each there was one, or more, detached fragment of bone. Two were wired on the sixth and fifteenth day respectively, and one was operated on at once. The first two resulted in the loss of the legs; the third patient recovered with a good leg. He believes this to be the best treatment of this fracture. Dr. Quenu[46] describes a case of incomplete inward subscaphoid dislocation, that is to say, displacement of the astragalus on the os calcis, but instead of the connection between the former and the scaphoid being severed, the latter was dragged along by the former and displaced. Extirpation of the scaphoid was effected, and the displacement of the astragalus on the os calcis reduced. About six weeks after the operation the patient left the hospital in a very satisfactory condition, being able to walk without limping.

[38] Medical Week, May 28, 1897. [39] Ibid, May 28. [40] N.Y. Med. Jour., August 7, 1897, p. 173. [41] Medical Week, June 4, 1897. [42] Medical Week, June 9, 1897. [43] Medical Week, June 18, 1897. [44] Clinical Journal, July 7, 1897, p. 161. [45] N.Y. Med. Jour., June 19, 1897, p. 827. [46] Med. Week, May 14, 1897.

### TECHNIQUE OF CEREBRAL SURGERY.

The limits to which surgical interference in cerebral conditions may legitimately go are gradually being laid down, and are apparently nearly reached. The more recent advances in the surgery of the brain have been chiefly in the direction of improving points in technique, with a view to diminishing the risks of operation—immediate and remote. In this matter American surgeons have recently been more active than those on this side, although on most points they carry us no further than did Macewen and Horsley in their pioneer work, and in many cases not so far.

**Asepsis.**—As regards the importance of avoiding sepsis in cranial operations all are agreed, and beyond the more modern advances in the use of aseptic, rather than antiseptic measures, there is little new to report. In cases of tumour, epilepsy, and such conditions as present unbroken skin, the ordinary antiseptic preparation of the scalp, instruments, and hands are generally understood and practised, while aseptic neutral salt solution and sterilised gauze are used for the interior of the cranium. For compound fractures and other injuries, abscesses, and infective conditions antiseptics have to be used even inside the skull. In a paper on the treatment of compound fractures of the cranium, Estes[1] lays great stress on the importance of those who render first aid in such cases contenting themselves with merely attending to urgent symptoms, and the application of such dressings as shall counteract or prevent infection, and the avoidance of all unnecessary handling, probing, or opening up of parts. A thorough and hopeful operation can only be done under hospital conditions, or such as nearly approach them. Much care is devoted to the removal of the hair, and the purification of the scalp, edges of wounds, and fragments of bone in the vicinity before further steps are taken. The best antiseptic for these purposes is 1·20 carbolic, followed by a free douching with neutral salt solution.

**Hæmorrhage.**—The control of hæmorrhage in cranial surgery is one of the most important problems that yet await solution. Bleeding from the scalp can usually be quite satisfactorily controlled by forceps, but some surgeons still prefer to use a band round the head. Summers,[2] for example, passes a strong elastic band round the forehead and occiput; and Weir[3] uses a rubber band with pads over the temporal fossæ. Parmenter[3] employs sutures so placed as to compress the vessels. Tiffany[3] uses forceps and a T-shaped bar.

Hæmorrhage from the dura mater may be controlled either by a ligature or by a fine catgut stitch passed round the vessel by means of a small needle; while that from the bone is best arrested by crushing with strong necrosis forceps or by plugging with gauze. In some cases a sterilised piece of wood pushing along the line of the bleeding point is efficient. It is, however, bleeding from large venous sinuses that gives most trouble. If the opening in the vessel is small a lateral ligature occluding the rent may be satisfactory, otherwise the best means available is plugging with gauze, the amount of pressure necessary being but slight. The only method available for preventing bleeding from the cortex is gauze packing. *Apropos* of bleeding in cerebral surgery Tiffany confirms an opinion, which has previously been expressed, that chloroform is the best anæsthetic to use in these cases; congestion of the head is much more marked with ether. He has observed during an operation that the change from ether to chloroform was followed by great diminution of the cerebral congestion.

**Opening the Skull.**—Outside of Britain a preference seems to exist for the osteoplastic method first introduced by Wagner in 1889, but most home surgeons still use the trephine. For the rapid and accurate cutting of osseous flaps many ingenious instruments have been devised. Wagner simply mapped out the bone flap by means of a groove cut with gouges, and then forcibly broke across the base of the flap, which, along with the overlying scalp, was thrown down. To get over the objection that the use of a hammer is said to produce symptoms of concussion, Cryer has contrived a spiral osteotome driven by a surgical engine. A small disc is first rapidly removed by a surgical engine, and through this opening a spiral osteotome is introduced, and rapidly cuts out a flap of the size and shape desired, provision being made for the protection of the dura mater by a metal plate, which depresses it in front of the advancing saw.

The breaking across of the base of the flap introduces a source of danger from splintering. To get over this, Müller includes in the flap only the outer table and diploe, and then removes the inner table with rongeur forceps. Keen does not use a dental engine, because he believes much skill is necessary to its safe employment; he prefers Pyle's chisels. Mixter[3] appears to favour the trephine, but insists on the mistake of using a large instrument. Much time is lost, and by a small disc rongeur forceps can be employed to enlarge to any extent with rapidity and safety.

Tiffany approves of performing these operations in two stages, the bone being opened as a first step, and, after a varying interval the soft parts being attacked. This, of course, is only applicable to chronic conditions. On this point Keen agrees with Tiffany, and would even go to the length of leaving the second stage for some months after the first.

The dura mater may be opened by a crucial incision, or, better still, in the form of a flap, cut some distance in from the bone to permit of subsequent suturing.

The skull having been opened, the interior may be explored. For the purpose of detecting the consistence of any mass inside the finger is the most reliable guide. An exploring needle may go through a neoplasm without giving any indication of its presence.

The question of drainage depends very largely on the condition for which the operation has been undertaken. If for compound comminuted fracture, meningitis, or suppuration, of course it is imperative. If for epilepsy, tumour, &c., much will depend on the amount of intercranial tension and the faith the surgeon has in his asepsis.

**Closure of the Skull.**—On this question there is much diversity of opinion, some authorities believing that no necessity exists for using more than the scalp as a covering, while others take great pains to effect closure either by bone or some foreign material. It has been frequently observed that bone discs replaced are apt to die and be thrown off as sequestra. This is doubtless the result of sepsis, but in other cases, while no infection has taken place, it has been found that the bone in course of time disappeared and was replaced by fibrous tissue.

Keen is of those who have never met with trouble after replacing bone, although in one case treated by his assistant the bone lost its vitality.

Apart from the question of a strong covering for the brain, it is of importance to prevent adhesions forming between the scalp and the dura mater or cerebral substance, as such adhesions are not infrequently the exciting cause of Jacksonian epilepsy. Many attempts have been made from time to time to prevent this by inserting between the scalp and the underlying tissues some inert, aseptic substance such as celluloid, a sheet of silver, or gold foil.

Weir, Meyer, and others have employed celluloid, fitting it accurately into the deficiency in the skull, so as to prevent granulations passing along the sides. In one of Meyer's cases small perforations were left for drainage, and granulations grew through these and re-established adhesions. Mixter has used gutta-percha for this purpose. A very interesting case is reported by Philip James,[4] of Wellington, N.Z., in which, after trephining for traumatic epilepsy following a fracture of the skull, he prevented the re-formation of adhesions by means of a silver plate. At the time the author was unaware that similar attempts had been previously made. The injury was sustained ten or eleven years before the onset of the epileptic seizures, and had left a depressed cicatrix $4\frac{1}{2}$ in. long. Before the operation a wax impression was taken of the area, and from this a plaster model was made, and to it was fitted a thin plate of pure silver. At the operation, which consisted in freeing the cicatrix, this plate was fitted into the deficiency. Eighteen months later there had been no return of fits, and the silver plate caused no inconvenience.

Gold foil has been more extensively used for this purpose. Tiffany[3] prefers it, and has observed patients for two years after its introduction without any trouble arising. Estes[1] has also been successful with it, as has also Beach.[5] These surgeons took care that the gold foil was so placed as to be firmly fixed into the bone, and so prevent the growth of granulations past it. In the case of Beach, five years elapsed between the introduction of the gold and the reporting of the case. Summers'[2] experience of the method also gives favourable results.

Weir has employed gold foil twice, but has found that granulations may penetrate it; and Woolsey[6] has had a similar disappointment. In his case the gold was in paraffined paper, and he fears that in the process of sterilising it by heat, and removing the paraffin, the foil may have been torn. On reopening the wound several months after operation, he found that adhesions had re-formed.

[1] Lehigh Valley Med. Mag., Dec., 1896. [2] Med. Record, Ap. 24, 1897. [3] Med. Record, June, 1897. [4] Australasian Med. Gazette, May, 1897. Boston Med. and Surg Jour., Mar, 1897. Annals of Surg., May, 1897.

### SURGERY OF THE PERITONEUM, &c.

**A revised nomenclature** is proposed by Maylard[1]—the object being to be sure that a certain operation performed upon the œsophagus under a particular name, that name should, with merely a regional distinction, convey a clear conception of a like operation performed on the stomach, bowel, or rectum. (1) The affix -tomy: for operations which consist in opening some part of the alimentary tract for exploratory and other purposes, and its closure at the same operation (œsophagotomy, cæcotomy, &c.). (2) The affix -stomy: (*a*) for operations which consist in stitching some portion of the alimentary tract to the parietes and establishing a temporary or permanent opening (œsophagostomy, sigmoidostomy, &c.); (*b*) for operations which consist in establishing permanent openings between one segment of the canal and another (in these compound words the proximal segment should be named first (gastro-jejunostomy, ileo-ileostomy, &c.). (3) The affix -ectomy: for operations which consist in the removal of some part of the canal (gastrectomy, proctectomy, &c.). (4) After excision of any part of the canal its continuity may be established by—(*a*) end to end junction, (*b*) lateral approximation (the ends are closed and openings are made in the side of the apposing coils), (*c*) lateral implantation (one end is closed while the other is fixed into a lateral opening of the occluded portion). (5) The methods by which the parts are secured together after incision or excision of any part of the tract are known mostly by the name of the materials used, or the authors devising them (union by Czerny-Lembert suture, union by Murphy's button, &c.). (6) The affix -rhaphy: for operations which consist in lessening abnormally enlarged or dilated sections of the tract by suturing together folds of the visceral parietes (gastrorrhaphy, colorrhaphy, &c.). (7) The affix -plasty: for operations performed for stricture of some part of the canal, the stricture being divided in the long axis, while the parietial edges are united in the transverse axis, thus forming a wider channel (pyloroplasty, enteroplasty, &c.). (8) The affix -pexy: for operations which consist in fixing by suture to the parietes some abnormally dilated or movable segment of the canal (gastropexy, colopexy, &c.). (9) Artificial anus: for operations for diverting the whole contents of the intestine through an artificial opening; performed by bringing a loop of bowel outside the parietes or breaking the continuity of the canal by section, and fixing the proximal extremity to the parietal incision (ascending colonic anus, sigmoid anus, &c.).

**Classification of Peritonitis.**—Senn[2] gives us the following syllabus of the acute forms: (1) Anatomical.—Ectoperitonitis, endoperitonitis, parietal peritonitis, and visceral peritonitis, viz., mesenteritis, epiploitis, perigastritis, peri-enteritis, perityphlitis, peri-appendicitis, pericolitis, perihepatitis, perisplenitis, pericystitis (urinary and gall-bladder), perimetritis, perisalpingitis, peri-oöphoritis, pelvic peritonitis, diaphragmatic peritonitis. (2) Etiological. — Traumatic peritonitis, idiopathic peritonitis, perforative peritonitis, metastatic peritonitis, puerperal peritonitis, peritonitis infantum, fœtal and intra-uterine peritonitis, peritonitis neonatorum. (3) Pathological.—Diffuse septic peritonitis, putrid, hæmorrhagic, suppurative, serous, and fibrino-plastic peritonitis. (4 Bacteriological. — Streptococcus infection, staphylococcus, pneumococcus, bacillus coli communis, gonococcus, and tuberculous infection. (5) Clinical.—Ectoperitonitis, general septic peritonitis, perforative, circumscribed, hæmatogenous, visceral (see under anatomical), pelvic, puerperal, and sub-diaphragmatic peritonitis.

**General Septic Peritonitis.**—McCosh[3] is opposed to the use of opium as a routine measure, and thinks that better results are obtained by the use of cathartics. On account of incessant vomiting these, however, cannot be administered by the mouth; and when given by the rectum seem almost valueless. For these reasons he has for some years employed the intra-intestinal injection of sulphate of magnesia and other cathartics. His method of operation in these cases is as follows: (1) Chloroform is employed as the anæsthetic. (2) A free incision is made, generally five or six inches in length, varying in situation according to the organ which has excited the peritonitis, and the patient is turned on the side to facilitate the escape of the purulent fluid. (3) As a rule the intestines are allowed to escape from the abdominal cavity into hot towels held in the hands of assistants. Their return is aided, if necessary, by opening the ileum and allowing gas and fæces to escape—the openings being closed by Lembert's sutures. (4) The cause (appendix, tumour, perforation) of the peritonitis is removed. (5) The intestines and peritoneum are thoroughly irrigated with hot normal salt solution. The temperature of the solution is 110° or 112° F. A considerable amount of the salt solution is allowed to remain in the abdominal cavity for the purpose both of stimulating the heart and of favouring intestinal drainage. (6) Sulphate of magnesia is injected, by means of a hollow needle attached to an aspirator, as high up in the jejunum or ileum as possible. A saturated solution containing between one and two ounces of the salt is used. The puncture is closed by a Lembert suture. (7) The peritoneal cavity is drained by four or more strips of sterile gauze thrust in different directions among the intestines, and at times a large glass tube is also inserted in the pelvis. (8) The abdominal wound is but partially closed by sutures. (9) After the return of the patient to bed, a ten-grain dose of calomel is given. Rectal stimulation is employed during the first 24 or 36 hours. Weir[4] thinks that cases where there is abundant lymph effusion in the peritoneal cavity give one a little more encouragement than those in which there is no lymph at all, or but very little. In the use of intra-intestinal injection of sulphate of magnesia he has not had the same successful results as McCosh. Abbe[5] thinks the intra-intestinal injection of cathartics is an important step, and also thinks that the application of ice over the abdomen after operation seemed to retard bacterial growth and diminish inflammatory action. He is opposed to the removal of lymph, which he regards as protective. Arsdale[6] has had two successful cases in which streptococcus anti-toxin was used. Finney[7] is a strong supporter of removing the lymph which forms in these cases. The peritoneal cavity, after the intestines have been drawn out, is thoroughly and systematically wiped with large pledgets of gauze wrung out of a hot solution of salt. The intestines, before being returned, are treated in the same manner, and he says the success of the operation depends upon the thoroughness with which this is done. Peyrot[8] has had recoveries after drainage of both iliac fossæ. Quenu[9] has had a similar success. Senn[10] is also in favour of the administration of cathartics by the mouth as a prophylactic measure or injected into the intestine after operative treatment, for he believes that free peristaltic movements of the intestine promote the absorption of septic material by the lymphatics. Antistreptococcic serum may be a valuable adjuvant in these cases, but cannot replace operative measures.

**Drainage of the Peritoneal Cavity** is believed by Clark[11] to be used too frequently, and he considers that in many cases a greater attention to detail in operation, with a greater reliance upon the ability of the peritoneum to eliminate infectious material, will overcome many difficulties which are now wrongly supposed to be obviated by drainage. For the prevention and removal of infection without the employment of drainage he lays down the following rules: 1. The hands must be thoroughly disinfected. 2. Hæmorrhage must be controlled. 3. Avoid bruising or otherwise injuring tissues. 4. Isolate the general peritoneal cavity during operation. 5. Preserve the peritoneum. 6. Conserve the body heat. 7. Avoid rupture of intra-peritoneal abscesses. 8. Irrigate the peritoneal cavity. 9. Promote absorption by saline infusions into the peritoneal cavity, followed by postural drainage. 10. If symptoms of infection arise after operation inject salt-solution infusions into the sub-mammary tissues.

**Tubercular Peritonitis** is cured, according to Gatti,[12] by a simple laparotomy, because as a result of the operation there is a fairly profuse exudate into the peritoneal cavity, which injures or destroys the bacilli, and, under certain conditions, leads to healing through a retrograde metamorphosis. Debove, Caubet, and Baylac[13] have employed puncture followed by lavage with a saturated hot watery solution of boric acid. They claim that by this means all the advantages of a laparotomy are obtained without exposing the patient to the dangers of that operation. Pure hot water is said to be even safer than the use of a liquid containing medicinal substances. Should there be much fluid in the peritoneal cavity before operation, it should be withdrawn by ordinary paracentesis before the lavage is attempted.

[1] Annals of Surgery, Sept., 1897. [2] Med. Rec. New York, Aug. 28 1897; and Amer. Journ. Med. Sci., Aug., 1897. [3] Annals of Surgery, June, 1897. [4] Ibidem, Aug., 1897. [5] Ibidem, Aug., 1897. [6] Ibidem, Aug., 1897. [7] New York Med. Rec., Aug. 14, 1897. [8] Med. Week, May 14, 1897. [9] Ibidem, May 28, 1897. [10] Brit. Med. Journ., Sept. 4, 1897. [11] Amer. Journ. of Obstet. and Dis. of Women and Child., Apr. and May, 1897; and Annals of Surg., Aug., 1897. [12] Am. Journ. Med. Sci., Aug., 1897; and New York Med. Journ., July 24 1897. [13] Therap. Gazette, Mar. 15, 1897.

